# A Comparison of Visual Assessment and Automated Digital Image Analysis of Ki67 Labeling Index in Breast Cancer

**DOI:** 10.1371/journal.pone.0150505

**Published:** 2016-02-29

**Authors:** Fangfang Zhong, Rui Bi, Baohua Yu, Fei Yang, Wentao Yang, Ruohong Shui

**Affiliations:** 1 Department of Pathology, Fudan University Shanghai Cancer Center, Shanghai, China; 2 Department of Oncology, Shanghai Medical College, Fudan University, Shanghai, China; H. Lee Moffitt Cancer Center & Research Institute, UNITED STATES

## Abstract

**Background:**

Ki67 labeling index (LI) is critical for treatment options and prognosis evaluation in breast cancer. Visual assessment (VA) is widely used to assess Ki67 LI, but has some limitations. In this study, we compared the consistency between VA and automated digital image analysis (DIA) of Ki67 LI in breast cancer, and to evaluate the application value of DIA in Ki67 LI assessment.

**Methods:**

Ki67 immunostained slides of 155 cases of primary invasive breast cancer were eyeballing assessed by five breast pathologists and automated digital image analyzed by one breast pathologist respectively. Two score methods, hot-spot score and average score, were used to choose score areas. The intra-class correlation coefficient (ICC) was used to analyze the consistency between VA and DIA, and Wilcoxon signed-rank test was used to compare the median of paired-difference between VA and DIA values.

**Results:**

(1) A perfect agreement was demonstrated between VA and DIA of Ki67 LI by ICC analysis (*P*<0.0001) in the whole cohort. A perfect agreement between VA and DIA of Ki67 LI was also showed in G2-G3, ER positive/HER2 negative cases. Average score and hot-spot score methods both demonstrated a perfect concordance between VA and DIA of Ki67 LI. (2) All cases were classified into three groups by VA values (≤10%, 11%-30% and >30% Ki67 LI). The concordance was relatively lower in intermediate Ki67 LI group (11%-30%) compared with high (>30%) Ki67 LI groups according to both methods. (3) All cases were classified into three groups by paired-difference (d) between VA values of hot-spot score and average score (d<5, 5≤d<10, d≥10) to evaluate the correlation between Ki67 staining distribution (heterogeneous or homogenous) and reproducibility of assessment. A perfect agreement was all demonstrated in three groups, and a slightly better Ki67 LI agreement between VA and DIA was indicated in homogenous staining slides than in heterogeneous staining ones. (4) VA values were relatively smaller than DIA values (average score: median of paired-difference -3.72; hot-spot score: median of paired-difference -9.12).

**Conclusions:**

An excellent agreement between VA and DIA of Ki67 LI in breast cancer was demonstrated in the whole mixed cohort, suggesting that VA and DIA both could be used to assess Ki67 LI in clinical practice. Average score and hot-spot score methods both demonstrated a perfect concordance between VA and DIA of Ki67 LI. The almost perfect agreement between VA and DIA was observed in high Ki67 LI cases, displaying a homogenous staining pattern. The consistency between VA and DIA was relatively low in intermediate Ki67 LI group. The heterogeneity of tumors may slightly affect the concordance between VA and DIA of Ki67 LI. Assessment of VA provides lower Ki67 values than DIA, the biological importance of these values are not known at the moment.

## Introduction

Uncontrolled proliferation is a key hallmark of malignancy. Ki67 is a proliferation-associated marker for tumors as it is expressed in all phases of the cell cycle, except G0 [1]. Ki67 labeling index (LI) is used as a proliferation marker and a predictive marker for response to chemotherapy, and is associated with prognosis in breast cancer [2, 3]. In 2011 [4], the 12^th^ St. Gallen Consensus Meeting suggested that the Ki67 LI was important for distinguishing between ‘‘luminal A” and ‘‘luminal B (HER2-negative)” breast cancer subtypes and advised adjuvant chemotherapy for luminal B but not for luminal A. Therefore, the standardization of the assessment of Ki67 LI is considered more important because of its impaction on clinical practice.

In 2011, the International Ki67 in Breast Cancer Working Group [5] published some recommendations for the assessment of Ki67 in breast cancer, aiming for better analysis, reporting, and use of Ki67. However, still no global guideline, with both reproducibility and objective standardization, has been established for Ki67 LI assessment in breast cancer. Visual assessment (VA) at a glance is now used to evaluate Ki67 LI in a considerable number of pathological institutions and laboratories, but there still are some limitations, for instance, the reproducibility of intermediate Ki67 LI and Ki67 LI in the moderately differentiated (G2) breast cancers in which Ki67 LI are crucial for making clinical decisions is relatively poor[6–10]. Automated digital image analysis (DIA) has been suggested as a potential method to improve the accuracy and inter-observer reproducibility in Ki67 assessment [11–12].

The aim of this study was to compare the consistency of VA and DIA of Ki67 LI in breast cancer, and to evaluate the application value of DIA in Ki67 LI assessment in clinical practice.

## Materials and Methods

### Case selection and Immunohistochemistry (IHC)

In our previous study [13], we have evaluated the interobserver concordance of VA of Ki67 LI in 160 cases of breast cancers. In this study, the same cohort was used except 5 cases which were unsuitable for digital image analysis because of the unsatisfied image quality. 155 cases of primary invasive breast cancer were randomly extracted from the pathology database of Fudan University Shanghai Cancer Center. All patients underwent surgery at the Cancer Center in 2012 without neoadjuvant chemotherapy. Because all cases in our study were randomly extracted from the pathology database, so all types of invasive breast cancers were included from grade 1(G1) to G3, ER negative and positive, PR negative and positive, HER2 negative and positive cases. Clinicopathological features of all cases were reviewed. All specimens were fixed with 10% neutral phosphate-buffered formalin and paraffin-embedded 4μm-thick slices of representative tumor blocks were stained with hematoxylin and eosin (H&E). Immunohistochemical analysis for Ki67 was performed on Benchmark XT system (Ventana, Tucson, AZ, USA), using MIB-1 antibody (dilution 1:100; Code M7240, Dako, Glostrup, Denmark).

### Visual assessment (VA)

**Fig 1:**
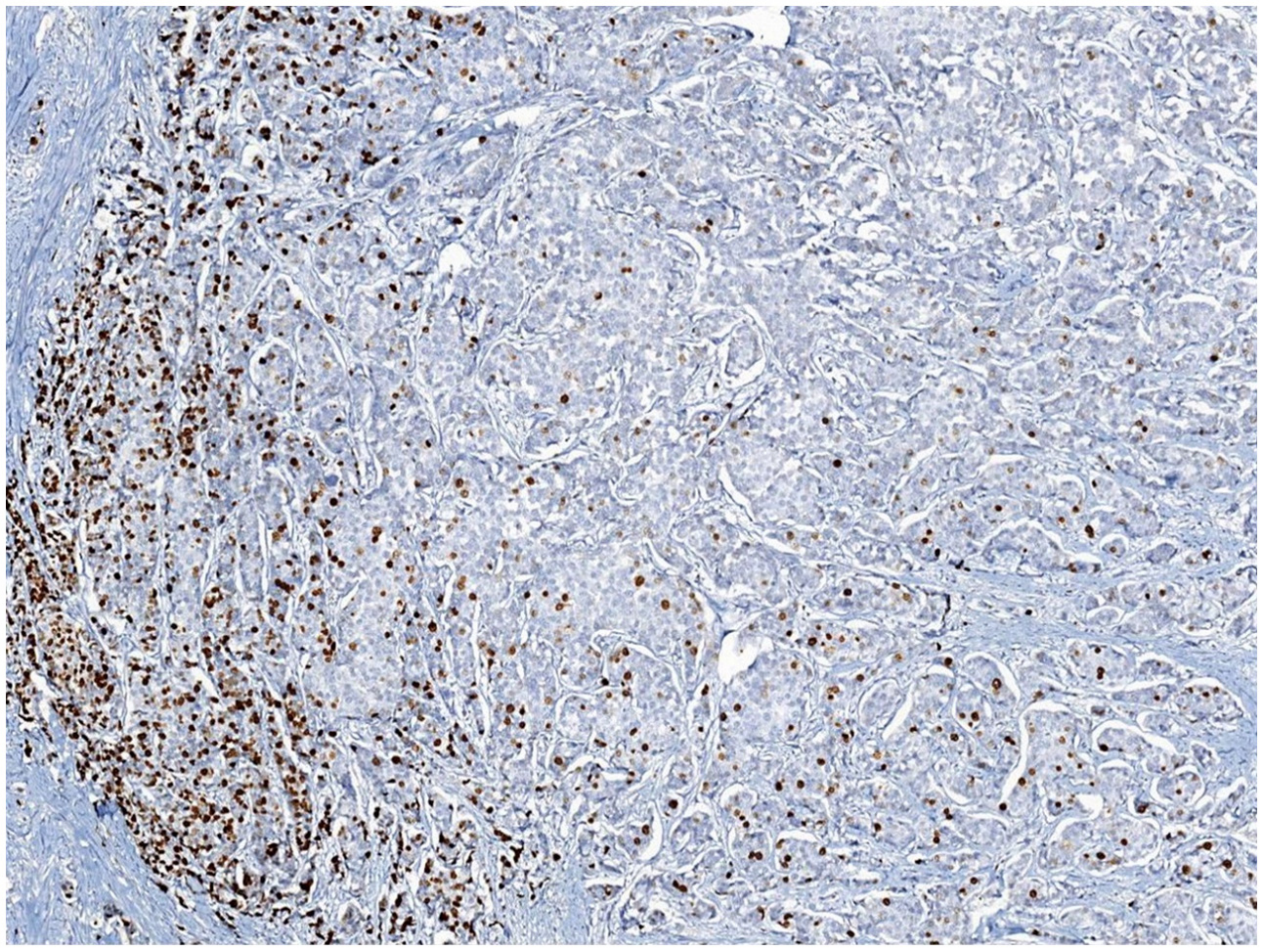
A heterogeneously stained case of Ki67. With a high Ki67 LI on the left and a low Ki67 LI on the right.

**Fig 2:**
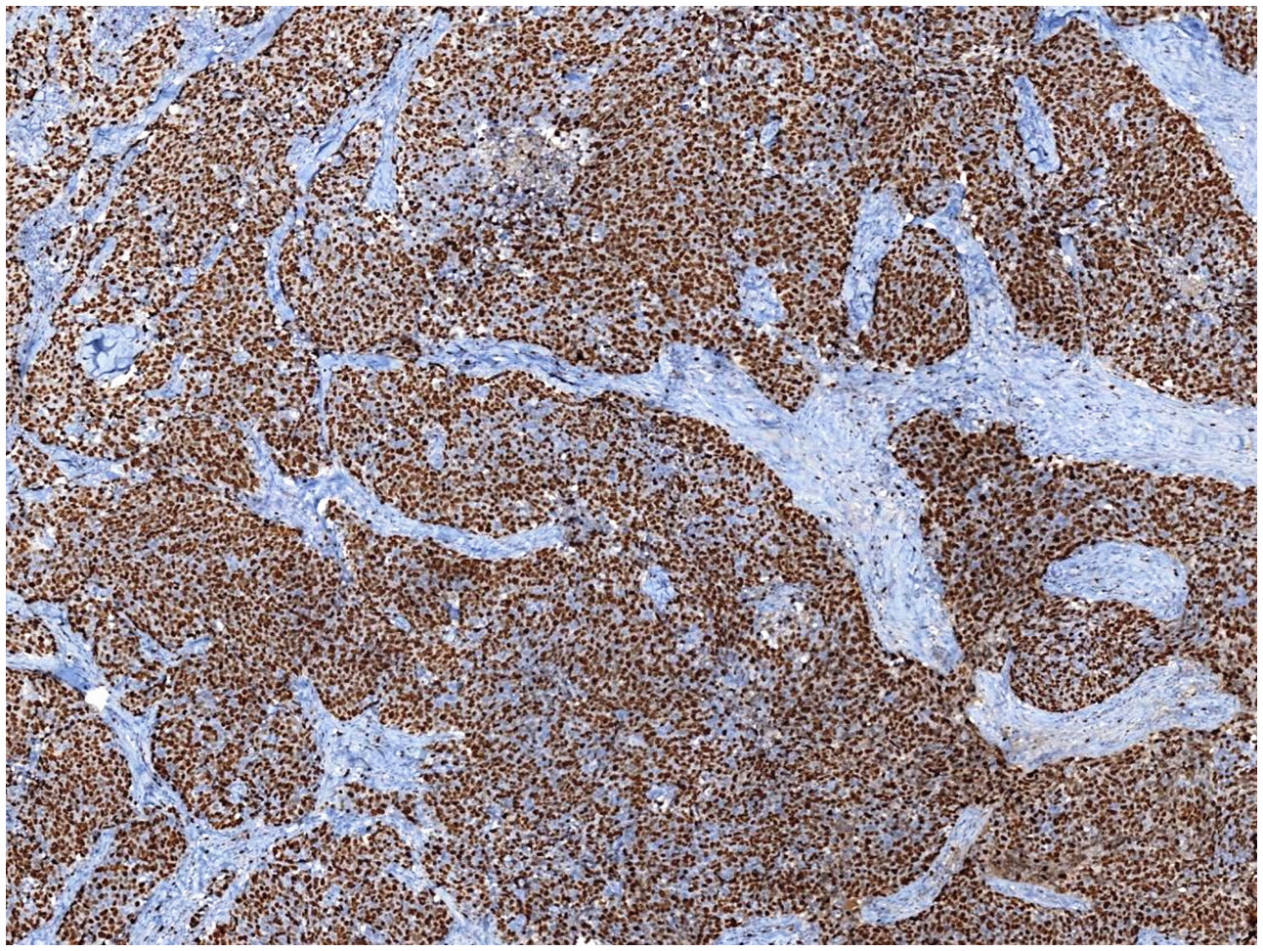
A homogenously stained case of Ki67. With a homogenous diffuse high Ki67 LI across the slide.

The Ki67 LI of all cases were visual assessed with specific instructions by five breast pathologists independently in a blinded manner. The mean values of five observers of each case were obtained as final VA values. Nuclear staining of any intensity was defined as Ki67-positive. The Ki67 LI was eyeballing scored for the percentage of positive tumor cells among all tumor cells in invasive tumor area at 10% intervals (i.e., 10, 20, 30, 40, 50, 60, 70, 80, 90, 100%). The whole slide was scanned at low-power microscopy first. At least three high-power (×40 objective) fields should be selected to represent the spectrum of staining seen on initial overview of the whole slide. In heterogeneously stained samples (Fig 1), each pathologist used two different methods to choose the scoring fields: (1) hot-spot score: the observers focused on the areas of hot spots, defined as areas in which Ki67 staining is particularly prevalent, and at least three independent areas were selected, and hot spots distributed in the invasive edge of the tumor must be included; (2) average score: the observers selected at least three independent areas including hot spots in an overall average assessment across the section. If the staining was homogenous (Fig 2), at least three randomly high-power fields were scored. The hot-spot score and average score were same in such samples. In our previous study [13], we have evaluated the interobserver concordance of VA of Ki67 LI among five observers in the same cohort. In this study, the data of Ki67 LI VA values of each observer were the same as in our previous study. The mean values of five observers of each case according to two score methods were recalculated as final VA values, which were used to compare with DIA values in this study.

### Digital Image Analysis (DIA)

**Fig 3:**
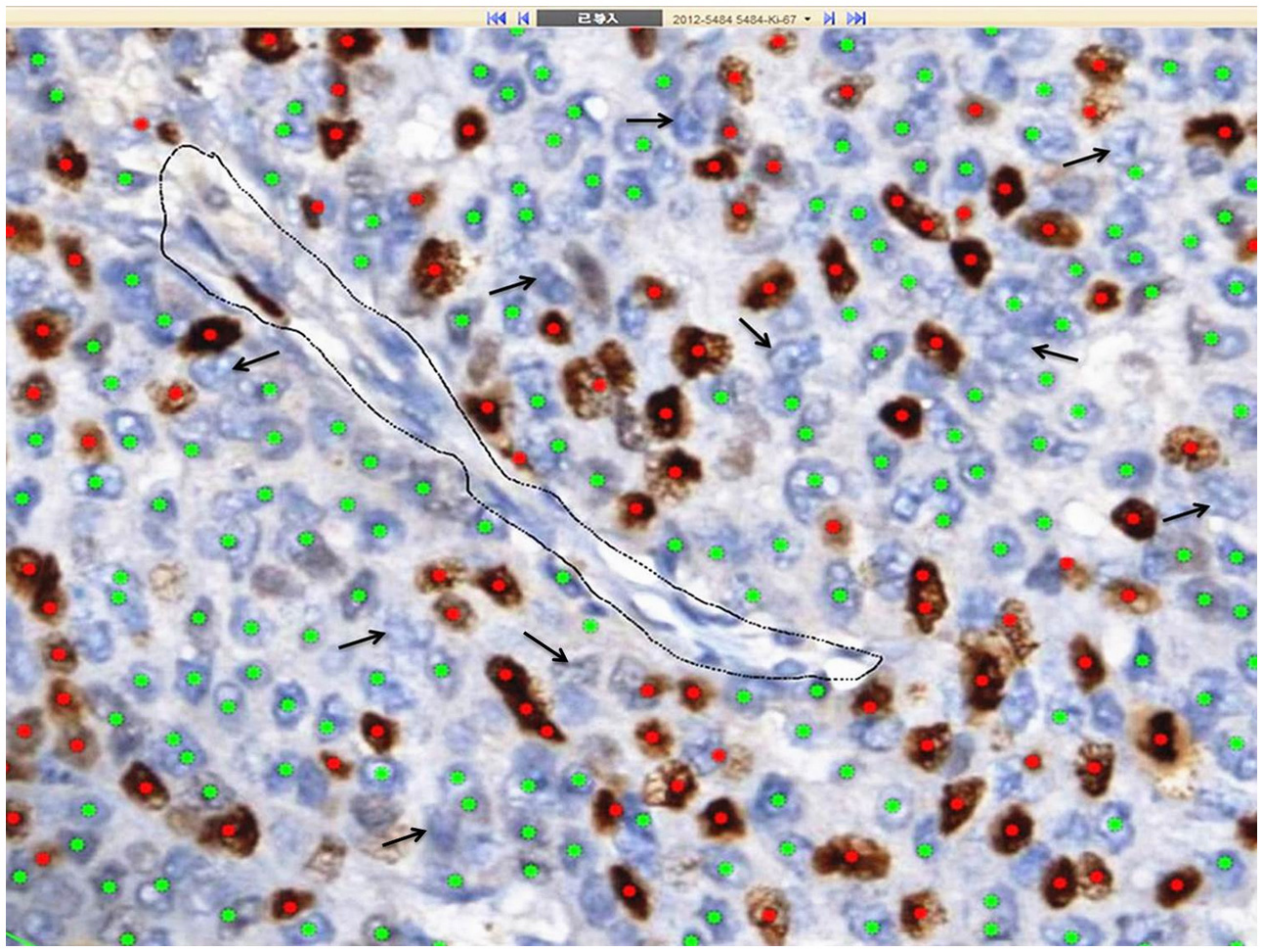
A score area was analyzed by DIA. Positive tumor cells were labeled with red dots and negative tumor cells with green dots. The areas surrounded by black lines were excluded. A few negative tumor cells weren’t recognized (black arrow).

**Fig 4:**
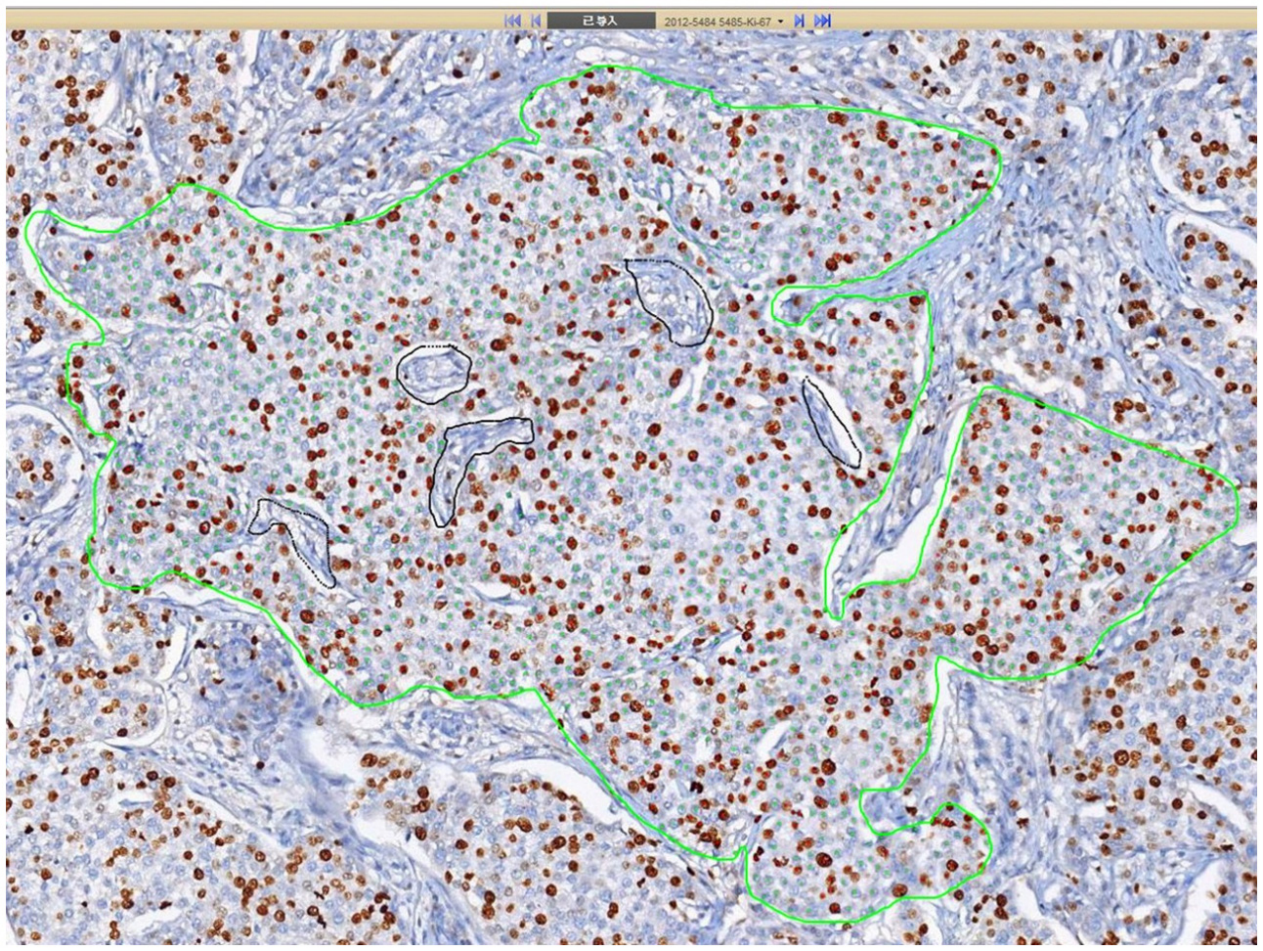
A selected area was to be analyzed by DIA. The scored area was surrounded by green lines and the mesenchymal components were excluded by black lines.

All the stained slides were scanned into digital slides by VENTANA iScan HT System Version 1.0 (Ventana Medical Systems, Inc, Sunnyvale, CA). The digital slides were analyzed by VENTANA Virtuoso Digital Pathology Image Analysis software Version 5.3 (Ventana Medical Systems, Inc, Sunnyvale, CA). The parameters of the software were set up as follows (Fig 3): large, round and brown stained nuclei were marked as red dots, representing positive tumor cells; round and not stained nuclei were marked as green dots, representing negative tumor cells; spindle mesenchymal cells were not marked. Firstly, the whole digital slide was viewed by another breast pathologist at a low magnification to choose the fields needed to be scored. Two different methods, with the same principle in VA, were used to select score areas: hot-spot score and average score including hot spots. At least five score areas would be selected to represent the spectrum of staining seen on initial overview of the whole digital slide. Each score area, containing about 1000 tumor cells, was surrounded by green lines, and the mesenchymal components were excluded by black lines (Fig 4). Ki67 LI of each selected field was analyzed by the digital image analysis software, and a mean value of each case was obtained according to two score methods.

### Statistical analysis

Statistical analysis was performed by the statistical software package SPSS 19.0 for Windows (SPSS Inc., Chicago, Illinois, USA). Intra-class correlation coefficient (ICC) was estimated with a 95% confidence interval (CI) using two-way mixed models to assess the consistency between VA and DIA assessment of Ki67 LI according to two score methods. Higher ICC usually indicates better consistency. There is no universally accepted standard criteria for the ICC, the following criteria, similar to the kappa coefficient were used here to aid interpretation [14]: 0.00–0.20 was interpreted as “slight correlation”; 0.21–0.40 as “fair correlation”; 0.41–0.60 as “moderate correlation”; 0.61–0.80 as “substantial correlation”; and >0.80 as “almost perfect correlation.” Two-sided P value less than 0.05 was considered to be statistical significant.

The difference between VA and DIA values was not accord with normal distribution (p for Kolmogorov-Simonov tests <0.01). Therefore, Wilcoxon signed-rank test was used to compare the median of paired-difference between VA and DIA values.

### Ethics statement

Our study was approved by Ethics Institutional Review Board of Fudan University Shanghai Cancer Center. The patient records/information was anonymized and de-identified prior to analysis.

## Results

### Clinicopathological data

All patients were female and ranged in age from 23 to 93 years, with a median age of 51. According to TNM stage, 44 cases were classified as stage I, 89 cases were stage II, and 22 cases were stage III. The tumors ranged from 0.5 to 5.0 cm in size, with a median diameter of 2.4 cm. 147 cases were diagnosed as invasive carcinoma of no special type (94.8%), and 8 cases (5.2%) were special subtypes of breast carcinoma (6 cases of invasive lobular carcinoma and 2 cases of mucinous carcinoma). Invasive tumors were grade 1(G1) in 6 cases, G2 in 80 cases and G3 in 69 cases. 118 cases were positive for estrogen receptor (ER), 104 cases were positive for progesterone receptor (PR), and 36 cases were positive for human epidermal growth factor 2 (HER2). 91 cases were classified as G2-G3, ER positive/ HER2 negative breast cancers.

### Evaluation of Ki67 LI

The detailed data of Ki67 LI scores of VA and DIA was provided in the S1 Dataset. The ICCs between VA and DIA of Ki67 LI according to two score methods were showed in Table 1. A perfect agreement was demonstrated between VA and DIA of Ki67 LI in the whole cohort of our study according to both score methods by ICC analysis. Average score method (ICC = 0.974, 95%CI 0.964, 0.981, P<0.0001) showed a slightly better consistency than hot-spot score method (ICC = 0.957, 95%CI 0.941, 0.968, P<0.0001).

**Table 1:** The ICC between VA and DIA of Ki67 LI in the whole cohort according to two score methods.

Score method	ICC(95% CI)	F-value	P-value
Average score	0.974(0.964, 0.981)	75.859	<0.0001
Hot-spot score	0.957(0.941, 0.968)	45.236	<0.0001

Since the value of Ki67 LI is more crucial for therapy decisions in G2-G3, ER positive/ HER2 negative breast cancers, a further analysis of consistency between VA and DIA of Ki67 LI in 91 cases of G2-G3, ER positive/ HER2 negative breast cancers were performed. It also showed a perfect agreement between VA and DIA of Ki67 LI in these cases by ICC analysis (Table 2). Average score method (ICC = 0.952, 95%CI 0.928, 0.968, P<0.0001) and hot-spot score method (ICC = 0.941, 95%CI 0.912, 0.961, P<0.0001) both showed a perfect consistency between VA and DIA of Ki67 LI.

**Table 2:** The ICC between VA and DIA of Ki67 LI in G2-G3, ER positive /HER2 negative cases according to two score methods.

Score method	ICC(95% CI)	F-value	P-value
Average score	0.952 (0.928, 0.968)	40.556	<0.0001
Hot-spot score	0.941 (0.912, 0.961)	33.019	<0.0001

Ki67 cutoffs ranging from 10% to 30% have been most widely used to classify patients into “Ki67 high” or “Ki67 low” risk groups for making clinical decisions. So in our study, all cases were classified into three groups (≤10%, 11%-30% and >30% Ki67 LI), stratified by VA values, according to two score methods respectively. In low Ki67 LI group (≤10%), it is incomparable because of the different assessment intervals of VA and DIA. The proportions of positive tumor cells were scored at 10% intervals by VA, so all the Ki67 LI were equal to 10% in this group. However, the Ki67 LI values around 10% scored by DIA were all exact values obtained from the digital image analysis software. In intermediate Ki67 LI group (11%-30%), a perfect correlation (ICC, 0.804 [95% CI 0.693, 0.877]) was showed by average score method and a substantial correlation (ICC, 0.678 [95% CI 0.480, 0.811]) was showed by hot-spot score method between VA and DIA. In high Ki67 LI group (>30%), a perfect correlation was both showed between VA and DIA according to average score (ICC, 0.945 [95% CI 0.911, 0.966]) and hot-spot score method (ICC, 0.903 [95% CI 0.855, 0.936]) (Table 3).

**Table 3:** The ICC between VA and DIA of Ki67 LI, stratified by VA values.

groups	Average score		Hot-spot score	
	N	ICC(95% CI)	N	ICC(95% CI)
≤10%	31	–	25	–
11%–30%	61	0.804(0.693, 0.877)	44	0.678(0.480, 0.811)
>30%	63	0.945(0.911, 0.966)	86	0.903(0.855, 0.936)

To evaluate the correlation between Ki67 staining distribution (heterogeneous or homogenous) and reproducibility of assessment, all cases were classified into three groups based on the paired-difference (d) between VA values of hot-spot score and VA values of average score (d<5, 5≤d<10, d≥10). The smaller the paired-difference between hot-spot score and average score, the more homogenous staining of Ki67 was indicated. ICCs between VA and DIA in three groups according to two score methods were evaluated respectively. A perfect agreement between VA and DIA was all demonstrated in three groups according to two score methods (Table 4). Two score methods both showed a perfect correlation in three groups: d<5 group (ICC, average score: 0.979 [95% CI 0.968, 0.986]; hot-spot score: 0.973 [95% CI 0.959, 0.982]), 5≤d<10 group (ICC, average score: 0.979 [95% CI 0.957, 0.990]; hot-spot score: 0.935 [95% CI 0.868, 0.968]), and d≥10 group (ICC, average score: 0.921 [95% CI 0.854, 0.958]; hot-spot score: 0.845 [95% CI 0.721, 0.916]). Smaller paired-difference meant more homogenous in staining. The ICC was observed to slightly decrease with increasing paired-difference, which indicated a slightly better Ki67 LI agreement between VA and DIA in homogenous staining slides than in heterogeneous staining ones.

**Table 4:** The ICC between VA and DIA of Ki67 LI, stratified by paired-difference between VA values of hot-spot score and average score.

groups	N	ICC(95%CI)for average score	N	ICC (95%CI) for hot spot score
d<5	80	0.979(0.968, 0.986)	29	0.973(0.959, 0.982)
5≤d<10	56	0.979(0.957, 0.990)	55	0.935(0.868, 0.968)
d≥10	19	0.921(0.854, 0.958)	71	0.845(0.721, 0.916)

d: paired-difference between VA values of hot-spot score and average score.

Wilcoxon signed-rank test was used to compare the median of paired-difference between VA and DIA values. VA values were relatively smaller than DIA values according to both score methods (average score: median of paired-difference -3.72, Z value -7.997, P<0.0001; hot-spot score: median of paired-difference -9.12, Z value -9.725, P<0.0001) (Table 5).

**Table 5:** The median of paired-difference between VA and DIA values of Ki67 LI.

Score method	median of paired-difference	Z-value	*P-value*
Average score	-3.72	-7.997	<0.0001
Hot-spot score	-9.12	-9.725	<0.0001

## Discussion

The potential clinical values of Ki67 LI in breast cancer have been recognized gradually in recent years. Several studies have showed high levels of Ki67 LI in breast cancer are associated with worse outcomes [2, 15, 16]. Selz et al. reported that Ki67 expression was a predictor of locoregional recurrence in breast cancer patients with negative lymph nodes after modified radical mastectomy [17]. In neoadjuvant treatment, Ki67 LI was associated with pathological response [3, 18]. However, there is still no standardized measurement methodology yet to evaluate Ki67 LI, which has hindered its use in clinical practice.

Manual counting of as many as 1000 tumor cells has often been recommended to evaluate Ki67 LI [5]. However, this method is tedious and labor intensive. Some studies highlighted the limitations of daily practice based on tumor cell counting and showed that the Ki67 LI values were significantly influenced by the inter-observer and intra-observer reproducibility [6–8, 19]. Visual assessment at a glance is a simpler method, which would be easier and faster. In the routine work of our department, visual assessment (VA) at 10% intervals is a main method to evaluate Ki67 LI in breast cancer. In our previous study [13], we evaluated the interobserver concordance of VA of Ki67 LI in breast cancer. A perfect agreement was demonstrated by VA as a whole, but the interobserver concordance of intermediate Ki67 LI in which most cutoffs are located for making clinical decisions was relatively low. Many studies showed a relatively poor consistency of Ki67 LI in the moderately differentiated (G2) breast cancers [6–10]. Recently, Varga et al’s study showed a moderate improvement in the inter-observer reproducibility of Ki67 LI in G2 breast cancer [20]. Automated digital image analysis (DIA) is a potential efficient method of Ki67 LI assessment, with benefits of increased capacity, precision and accuracy when compared to visual evaluation or counting [8, 9, 21–24]. In this study, we compared the consistency of VA and DIA of Ki67 LI in breast cancer. Our study showed a perfect agreement between VA and DIA of Ki67 LI in the whole cohort. Since the value of Ki67 LI in patients with ER negative, HER2 positive or G1 is much less relevant for therapy decisions, we excluded these cases from our cohort and performed a further analysis. A perfect agreement was also demonstrated in G2-G3, ER positive/HER2 negative cases. Our study indicated that VA and DIA both could be used to assess Ki67 LI in clinical practice.

Heterogeneity of Ki67 staining can occur across breast cancer cases, and which areas of the tumors should be scored is controversial. It has been an important reason of the poor interobserver concordance in Ki67 LI evaluation. The approach to selecting scoring areas varies across studies [5, 13, 19]. In this study, we adopted two different score methods, average score and hot-spot score, to evaluate the concordance between DIA and VA of Ki67 LI. As a result, two score methods both showed a perfect consistency between VA and DIA of Ki67 LI. Average score method (ICC = 0.974) showed a slightly better agreement than hot-spot score method (ICC = 0.957), but the difference was extremely small. In Varga et al’s study [20], a good intra-observer reliability was also demonstrated by all score methods on light-microscopy for Ki67 assessment.

Ki67 cutoff values, correlated with clinical decision-making in breast cancer, remain controversial and have not been clearly established yet [10, 25–27]. Multiple studies in early breast cancer showed that cutoffs ranging from 10% to 30% have been most commonly used [10, 25–29]. Many studies showed high interobserver variability among cases with intermediate Ki67 LI, the region in which most cutoffs are located for making clinical decisions [10, 13]. Computer-based image analysis may be a potential method used in the intermediate Ki67 LI groups [19]. In our study, we compared the consistency between DIA and VA in different Ki67 LI groups. It is incomparable because of the different assessment intervals of VA and DIA in low Ki67 LI group (≤10%). However, Ki67 LI is not crucial in decision-making of the treatment in this group. An excellent agreement between DIA and VA was showed in high Ki67 LI group (>30%). In intermediate Ki67 LI group (11%-30%), the group with obvious clinical significance, the consistency between VA and DIA was substantial (hot-spot score) to perfect (average score), lower than high Ki67 LI group, but was acceptable. Our study showed that DIA may be a potential method to evaluate the tumors with intermediate Ki67 LI, but further research should be conducted to evaluate the reproducibility of this method.

Ki67 staining was homogenous in some cases and heterogeneous in others. According to the paired-difference (d) between VA values of hot-spot score and average score, all cases were classified into three groups (d<5, 5≤d<10, d≥10) in our study. Smaller paired-difference suggested more homogenous in staining. A perfect consistency between VA and DIA was all demonstrated in three groups, and a slightly better Ki67 LI agreement was observed in homogenous staining slides than in heterogeneous staining ones. Our study indicated that the heterogeneity of tumors may slightly affect the consistency between VA and DIA of Ki67 LI.

Although the consistency between VA and DIA was very good by ICC analysis in our study, DIA values were relatively larger than VA values as a whole. The biological importance of these values isn’t known at the moment and further research is still needed. The main reason for this discrepancy between VA and DIA may be a bias in identification of tumor cells. DIA has the advantage of measuring a much larger number of cells and strong objectivity, as compared to human operator at the microscope. However, DIA also has some disadvantages compared with VA, such as the identification of tumor cells. In terms of VA, tumor cells are easily distinguished from lymphocytes or other stromal cells by observers. But sometimes DIA is unable to distinguish tumor cells from lymphocytes, such as a few Ki67-positive lymphocytes may be recognized as tumor cells, or a few negative tumor cells may be misinterpreted as lymphocytes and not be recognized (Fig 3), which would lead to an increasing Ki67 LI value. Double stains may be used as a potential method to highlight tumor cells and to improve the accuracy of DIA in Ki67 evaluation in future studies [30].

In conclusion, our study showed an excellent agreement between VA and DIA of Ki67 LI in breast cancer in the whole mixed cohort, suggesting that VA and DIA both could be used to assess Ki67 LI in clinical practice. Average score and hot-spot score methods both demonstrated a perfect concordance between VA and DIA of Ki67 LI. The almost perfect agreement between VA and DIA was observed in high Ki67 LI cases, displaying a homogenous staining pattern. The consistency between VA and DIA was relatively low in intermediate Ki67 LI group, and further research should be conducted to evaluate the reproducibility of DIA in this “gray zone” group. The heterogeneity of tumors may slightly affect the consistency between VA and DIA of Ki67 LI. Assessment of VA provides lower Ki67 values than DIA, the biological importance of these values are not known at the moment.

## Supporting Information

S1 Dataset The detailed data of Ki67 LI scores of visual assessment and automated digital image analysis. (XLSX)
